# COVID-19 Vaccine Hesitancy in Pakistan: A Mini Review of the Published Discourse

**DOI:** 10.3389/fpubh.2022.841842

**Published:** 2022-05-31

**Authors:** Sumaira Khalid, Bilal Ahmed Usmani, Sameen Siddiqi

**Affiliations:** ^1^Epidemiology and Biostatistics Section, Community Health Sciences, Aga Khan University, Karachi, Pakistan; ^2^Health Policy and Management Section, Department of Community Health Sciences, Aga Khan University, Karachi, Pakistan

**Keywords:** COVID-19, vaccine hesistancy, population health, meta-analysis, review

## Abstract

This minireview provides a summary of the main findings, features, as well as limitations and gaps in the current epidemiologic research on COVID-19 vaccine hesitancy (VH) in Pakistani population. For this purpose, data on VH studies were extracted from January 2020 to October 2021, using a systematic review and meta-analysis approach. Literature review and other narrative studies were excluded. There exists a significant heterogeneity in the reported vaccine hesitancy in the population (pooled estimates from random-effects meta-analysis: 35% (95% CI, 28–43%). However, none of the co-variables included in the studies explained the observed variance/heterogeneity in the moderator analysis models. In this minireview and critical appraisal of current VH research, we conclude that an in-depth analysis of COVID-19 vaccine hesitancy in a representative sample of Pakistani population is crucial to measure the magnitude of VH as well to explore and identify the determinants of VH in Pakistani population. This is an important step toward informing intervention and policy design and to address this issue at its root cause. To this end, focused, methodologically robust and hypothesis-driven VH research is needed using a wide range of co-variables to support a detailed coverage of the individual and environmental level VH attributes.

The COVID-19 pandemic witnessed a surge in community health research (CHR) in Pakistan on a range of topics concerning the infection, its spread, and the potential implications for health policy and interventions. Early research focused on exploring knowledge, attitudes, and practices pertaining to prescribed preventive measures and estimation of the changing burden of COVID-19 infections in the population. After the arrival of COVID-19 vaccine, the discourse, however, shifted toward the uptake and hesitancy of vaccine in the Pakistani population. Vaccine hesitancy (VH), as defined by the WHO, is a complex and context-specific concept revolving around three main pillars: complacency, confidence, and convenience. VH is not a novice concept in Pakistan's healthcare landscape; therefore, considering the historic polio vaccination challenges, the number of CHR studies exploring COVID-19 vaccine hesitancy and acceptance, and the effect of the government's vaccine mandate in Pakistan has risen exceedingly.

Vaccine hesitancy-community health research bears implications on much larger levels including public health measures, future research directions, and policy design and implementation. VH-CHR serves three major goals: (1) measures the magnitude of the VH issue, (2) explores and identifies the determinants of the VH issue, and (3) offers an action-oriented narrative, cognizant of the rich context and features of VH in the population, to inform public health messaging, interventions, prospective research, policy design, and implementation (1–3). We suggest that the existing VH-CHR in Pakistan falls short of all the three goals, and despite the increasing number, the overall findings from individual VH studies remain inconclusive, especially in estimating (1) VH magnitude across different population subgroups, (2) determinants of different VH proportions across subgroups (e.g., small vs. large provinces), and (3) influential features and areas for a long-term action plan.

To capture a holistic account of the published VH-CHR in Pakistan, a total of 323 studies were extracted using the systematic review and meta-analysis approach, starting from January 2020 to October 2021. Of the 323 studies on COVID-19 vaccination, 72 were selected for abstract screening, 19 for full-text screening, yielding 10 eligible studies (i.e., original epidemiologic research articles) that addressed VH in Pakistani populations including 9 cross-sectional survey studies (4–12) and 1 quasi experiment-based study (13). One of the eligible studies did not report VH proportion as their primary outcome and was excluded from quantitative analysis. Assuming a significant level of heterogeneity across the studies, a random-effects meta-analysis (RMA) model was used to summarize the findings of the included studies.

## Analysis of Main Findings

**Figure 1:**
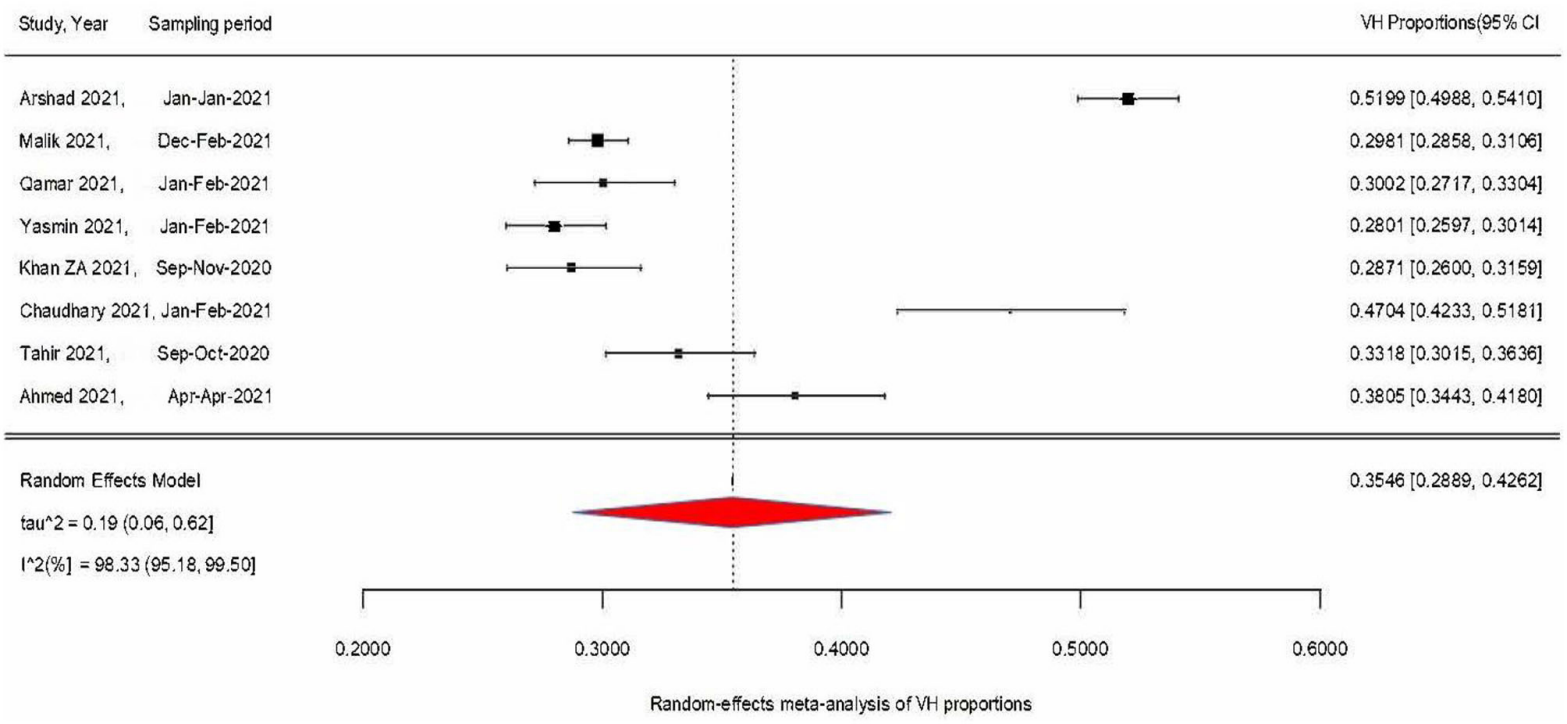
Meta-analysis of vaccine hesitancy proportions (Jan 2020 to Oct 2021).

**Figure 2:**
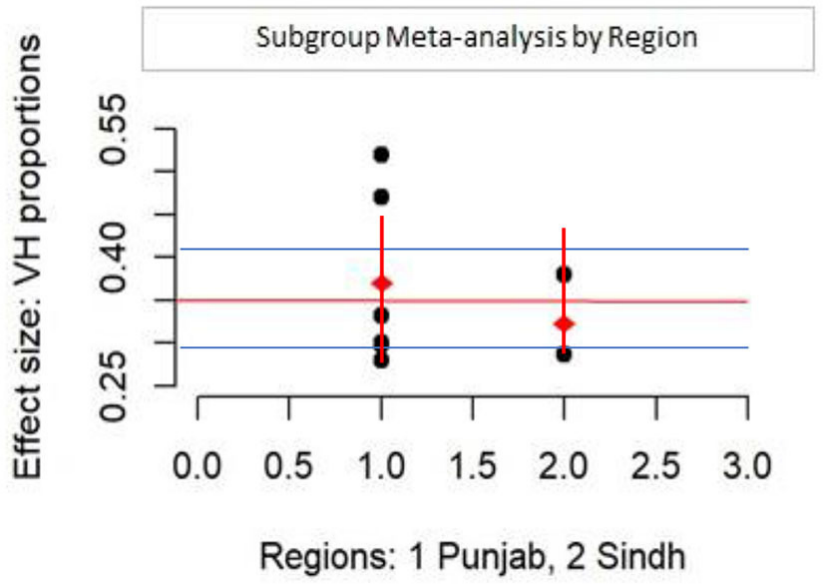
Subgroup analysis by dominating region.

Figure 1 condenses findings from the RMA model using a forest plot of the weighted VH proportions (WVPs) of the included VH studies and the overall VH proportion (pooled estimate) derived from the meta-analysis, i.e., 35% (95% CI: 29–43%, *p* < 0.0001). Only two studies' WVPs exceeded the measured pooled estimate, while 6 other WVPs were at or below the pooled estimate. Test for heterogeneity (*I*^2^) was 98% (95% CI: 95–99, *p* < 0.0001), indicating a statistically significant presence of variation/heterogeneity across WVPs. To identify the co-variables explaining the observed variance across VH proportions, meta-regression moderator analyses were conducted on available covariates, namely, age, sex, region, study type (online vs. mixed method), and study period (Table 1). Table 1 shows that none of the covariables were significant and that their contribution to explaining the observed variance/heterogeneity was unremarkable and statistically insignificant (explained by Q statistics, and *p*-values). The majority of study respondents were from Punjab province (*n* = 6) and to assess the difference in VH proportions for Punjab vs. Sindh province, a subgroup analysis was performed (Figure 2). The pooled VH estimate for Punjab studies (36%, 95% CI: 28–45) was higher than both the overall pooled estimate and that for Sindh (33%, 95% CI: 25–43). Test for heterogeneity was significant for the Punjab subgroup (Punjab (*n* = 6), *I*^2^ = 98.75%, Q = 400.49), while for Sindh, despite a high *I*^2^ (93.67%, Q = 15.81; *p*-values <0.01), it remained inconclusive on account of small subgroup size (*n* = 2). The difference across provinces was found to be statistically insignificant in the moderator analysis (Q = 0.22, *p*-value = 0.64).

**Table 1:** Meta-regression analysis: test for moderators.

**Moderator**	**Estimate, *P*-value**	***Q*, *P*-value**
**Age in Yrs**.		1.4736, 0.9612
Mean 23–30	0.0073, 0.6729	
**Majority sex**		1.2030, 0.5480
Female vs. NA	0.0843, 0.5657	
Male vs. NA	−0.0417, 0.7662	
**Study period**		1.3606, 0.9286
Sep to Oct-2020	0.0302, 0.9014	
Sep to Nov-2020	0.0600, 0.8039	
Dec to Feb-2021	0.0534, 0.8311	
Jan to Jan-2021	−0.0913, 0.6315	
Jan to Feb-2021	0.0191, 0.9226	
Apr to Apr-2021	Reference	
**Study type**		0.0120, 0.9130
Online Vs. mixed	−0.0147, 0.9130	

Vaccine hesitancy research is crucial to provide in-depth analysis and insight into the population and environmental level determinants of the increasingly important VH issue in the country. The overall finding of relatively low vaccine hesitancy vs. vaccine acceptability in the general population (pooled estimates, respectively, 35 vs. 65%) suffers from a high degree of bias, mainly inherited from research design, underlying framework, selection and sampling, and coverage of covariates. For example, the exclusively self-administered online-based design of the studies resulted in the recruitment of a disproportionately younger study sample (aged between 18 and 30 years, an average of 23 years). This rendered “age,” a potential predictor for VH in any population, an unproductive covariate. The resulting under representation of middle-aged and older adults in the current VH-CHR compromised our understanding of VH determinants and other correlates in the middle-aged and older population subgroups. Similarly, the majority of the study participants were University students, unmarried, female, had access to computers, and belonged to the middle and upper-middle socioeconomic groups. It would be exceedingly helpful to explore the relationships between rurality, ethnicity, and VH; however, the majority of the studies drew data from predominantly urban regions of Punjab and Sindh, leading to a glaring under-representation of smaller, dominantly rural provinces (e.g., Khyber Pukhtunwa, Balochistan, and Gigit-Baldistan). Similarly, those married and those with children make an important population in which to explore VH. The representation of these subgroups was highly under-whelming in current VH studies.

Similarly, other major drivers of VH, such as lower education, poor healthcare access, perceived health status, quality of life, unemployment, and homelessness, remained under-explored. Lastly, as alluded above, the frequently used self-administered online cross-sectional survey design has led to oversimplification and binarization of VH, an issue deeply rooted in ethnocultural, religious, and socioeconomic complexities.

Table 2 summarizes the major themes of the included VH studies. These themes stem from three fundamental yet intersecting elements: (1) conspiracy and religious beliefs, (2) education on COVID-19 and vaccine safety and efficacy, and (3) lack of trust. Although some of the findings suggest that the government's COVID-19 vaccine mandate might have helped in getting people vaccinated, research indicates that it is not a stand-alone driver of vaccine acceptance and compliance in the population. The repulsion of the deep-seated VH behavior and related concerns in the population would take a detailed multilevel analysis aimed at identifying the modifiable factors of VH in the individual (e.g., age, sex, educational attainment, employment type/status, and access to healthcare/insurance) and environmental (e.g., rurality, healthcare infrastructure, healthcare inequities, and public health policies) levels. For example, the demographic attributes of those with insufficient knowledge of efficacy would likely be different than those of believers in natural immunity dogma. Similarly, a subgroup that believes that a vaccine is perhaps not widely available or is available at a high cost might differ in features from a subgroup that believes the vaccine is toxic or ineffective. Identification of those differentiating features, which could come either from the individual or environmental level or both, is imperative for the purpose of targeted intervention and informed policy changes.

**Table 2:** Qualitative summary of the findings from VH studies (*N* = 9).

**Major themes**	***N* (%)**
Conspiracy beliefs/religious beliefs	3 (33)
Vaccine unavailability	2 (22)
Non-healthcare workers/care providers	2 (22)
Insufficient knowledge on efficacy and effectiveness	4 (44)
Toxicity/adverse effects/side-effects	3 (33)
Denial vs. perceived fear of COVID	2 (22)
Natural immunity dogma	2 (22)

In addition to the aforementioned methodological gaps, the conceptual framework of current VH-CHR has largely been drawn on the acute VH models (i.e. short-term and reactive vs. long-term and proactive VH models) for example (1) reinforcement of vaccine mandate, (2) fear-based public health messaging, (3) calling on other non-public health bodies for engagement e.g., print, live, and online media, and (4) calling on direct care providers and healthcare practitioners to take the lead. Therefore, the current VH-CHR discourse is dominated by the notion of approximating acute VH models (points 1–4, above) as some panacea for the ingrained VH issue in Pakistan. This undermines the need for a proactive strategy that is necessary for developing long-term, sustainable, and multisectoral public health efforts. One solution could be to balance the narrative by incorporating acute and long-term factors, e.g., the momentum and advocacy of the government-imposed punitive measures and mandates could be balanced out by making inroads in quality research aimed at identifying modifiable determinants, barriers, and facilitators of VH in different ethno sectoral subgroups in Pakistan to arrive at dependable long-term solutions.

The ideological framework could also use principles of compassion and empowerment, elements that form the very crux of public health practice, by adjusting the tone and framing of the current VH-CHR away from public blaming (14). In part, this can be achieved by identifying and acknowledging the public's VH beliefs and concerns and by attempting to explore the prodromal factors rooted in the system and environmental levels.

For example, only one study included in our thematic analysis (Table 2) had discussed VH in the context of mistrust that was generated by frequently changing narratives of international public health agencies (13). Similarly, the phase-wise vaccine distribution and its performance in flattening the epidemic curve remain understudied in the context of the Pakistani population, a relatively young population suffering from drastic healthcare inequities. Evidence is scarce but nevertheless exists, e.g., a recent modeling study evaluated the health impact and cost-effectiveness of COVID-19 vaccine distribution in Sindh; their models demonstrated that prioritizing vaccination for elders (>65 years) could only be effective in populations with high proportions of elders or in places where the vaccination had a significantly high impact on curbing the transmission of the pathogen (15).

Long-standing VH-CHR in other countries informs that majority of individual-level VH determinants are close correlates of environmental-level factors. Countries with a strong public health infrastructure were able to address the arising VH in their populations at a much faster rate. In Pakistan, the historic lack of public health leadership remains a huge environmental-level effect mediator of VH in the nation. The resulting void is consequently providing a fertile ground for breeding theories of fear, conspiracy, and mistrust in the nation. In addition to VH, other public health challenges facing Pakistan, e.g., the alarmingly rising antibiotic pan-resistance, is also in part due to the lack of strong health infrastructure, causing unregulated clinical and dispensing practices. By mid-century, the burden of non-communicable diseases will rise inexplicably and be fuelled by an increasingly aging population. Coupled with the emergence of novel communicable diseases, it is imperative that Pakistan's major health agencies do not solely rely on policies that are born out of acute necessity and, hence, die out when the necessity goes away. In the past and during the ongoing pandemic, independent health institutions and international organizations have gathered and have partnered with government agencies to form a consortium for addressing major public health issues at hand. However, such efforts were short-lived and, perhaps because of a lack of ownership, did not translate into full-scale, long-term, and dependable solutions. We argue that in addition to addressing deep-seated public health challenges, strong public health infrastructure in the country will also foster the quality of CHR activities by (1) streamlining and sustaining public health programs and research activities, (2) resourcing and allocating large funds to high-priority and under-explored areas, and (3) provisioning early career researchers with sufficient funds to support capacity building and training activities in public health institutions nationwide.

Recent systematic and scoping reviews on COVID-19 VH and its determinants in other countries have indicated that despite low vaccine acceptance rates among Middle Eastern, African, and certain European countries (16), VH determinants and their contextual factors vary widely within and across different populations (17, 18). For example, a comprehensive review on determinants of VH in high-income countries has reported female sex, younger age, ethnic minority, and lower educational attainment to be associated with increased VH in addition to lack of trust, history of a flu shot, and absence of any chronic conditions; this may very well indicate possibilities of interactions and effect modification between different VH covariates (17). In this study, we have attempted to summarize the current, most updated VH-CHR, and its strengths and limitations. The major strength of our study is the use of rigorous systematic review and meta-analysis methodology to summarize the extant research on this topic. There are some limitations, however, that remained in our study. For example, the quality of individual studies included in our meta-analytic model could not be adequately assessed using a standard tool such as the Newcastle-Ottawa Scale for assessing the quality of observational studies in meta-analyses. The overall quality of the included studies was not very high, and the number of parameters was very limited. The application of meta-regression for moderator analysis did not yield any substantial findings for this very reason. In the future, we would like to extend this study by including studies from other countries with a socioeconomic profile similar to that of Pakistan. Other limitations include small sample size and a lack of detailed thematic analysis. Furthermore, we recommend that the existing VH-CHR narrative be revisited to increase its coverage, functionality, and rigor. This can be achieved by employing a mixed-methods research approach and by taking into account the diverse demographic features and contours of the Pakistani population. In addition to using better sampling techniques, a larger and representative sample size, and better coverage of covariates, we also recommend future studies on VH to use validated ethnographic and cross-cultural research models to undertake high-quality research with a substantial value for the public health practice and policy in Pakistan.

## Author Contributions

SK: conception of the study, drafting and revision of the work, analysis and visualization, final approval, and agreeing to the accuracy of the work. BU and SS: critical revision of the work, final approval, and agreeing to the accuracy of the work. All authors have read and approved the final manuscript.

## Conflict of Interest

The authors declare that the research was conducted in the absence of any commercial or financial relationships that could be construed as a potential conflict of interest.

## Publisher's Note

All claims expressed in this article are solely those of the authors and do not necessarily represent those of their affiliated organizations, or those of the publisher, the editors and the reviewers. Any product that may be evaluated in this article, or claim that may be made by its manufacturer, is not guaranteed or endorsed by the publisher.
